# GRK5 functions as an oncogenic factor in non-small-cell lung cancer

**DOI:** 10.1038/s41419-018-0299-1

**Published:** 2018-02-20

**Authors:** Li-Ping Jiang, Song-Qing Fan, Qiu-Xia Xiong, Yong-Chun Zhou, Zuo-Zhang Yang, Gao-Feng Li, Yun-Chao Huang, Meng-Ge Wu, Qiu-Shuo Shen, Kun Liu, Cui-Ping Yang, Yong-Bin Chen

**Affiliations:** 1 Key Laboratory of Animal Models and Human Disease Mechanisms, Kunming Institute of Zoology, Chinese Academy of Sciences, Jiao Chang Dong Lu 32, 650223 Kunming, China; 2 Kunming College of Life Science, University of Chinese Academy of Sciences, 100049 Beijing, China; 3 Department of Pathology, The Second Xiangya Hospital, Central South University, Changsha, Hunan China; 4 Kunming Medical University, 650223 Kunming, China

## Abstract

Lung cancer is the leading cause of cancer-related deaths worldwide, and non-small-cell lung cancer (NSCLC) accounts for about 80% of all cases, which is the major subgroup of lung cancer. G protein-coupled receptor kinase 5 (GRK5) has been demonstrated to play pivotal roles in both development and progression of several pathological conditions including cancer. Here, we found that GRK5 expression was significantly increased in 539 NSCLC cancerous tissues than that in 99 normal non-cancerous tissues by immunohistochemistry analysis; we also showed intensive higher positive staining percentage in female and adenocarcinoma (ADC) NSCLC patients than that in male and squamous cell carcinoma (SCC) patients, respectively. In addition, GRK5 high expression NSCLC patients had a worse overall survival rate than the low expression patients. We provided evidence showing that both the mRNA and protein expression levels of GRK5 were increased in NSCLC cancerous cell lines (GLC-82, SPC-A-1, H520, H838, H358, A549, and H1299) comparing with that in normal human bronchial epithelium cell line (BEAS-2B), and identified many GRK5 mutations in NSCLC cancerous tissues. In addition, we found that depletion of GRK5 inhibited NSCLC cancerous cell proliferation, migration in vitro, and xenograft tumor formation in vivo. Furthermore, GRK5 knockdown promoted cell cycle arrest at G2/M phase and induced cellular apoptosis. In summary, our data reveal an oncogenic role of GRK5 in NSCLC progression, indicating that GRK5 could be used as a new therapeutic target in future.

## Introduction

Lung cancer is a leading cause of cancer-related deaths worldwide^1,2^. Non-small-cell lung cancer (NSCLC) accounts for approximately 80% of lung cancer cases, which are divided into three classes including squamous cell carcinoma (SCC), adenocarcinoma (ADC), and large-cell carcinoma^3^. Although many therapies have been developed to improve the outcomes, the median survival time of patients with NSCLC is less than a year after diagnosis^2^. Combination usage of the epidermal growth factor receptor tyrosine kinase inhibitor (EGFR-TKI) has been more effective to the subset of lung cancer patients carrying L858 or exon 19 deletion EGFR mutations^4^. However, the emergence of both primary and required resistance to EGFR-TKIs has led to the development of new generations of EGFR-TKIs or combination therapy targeting to other targets.

G protein–coupled receptor kinases (GRKs) are a family of serine/threonine kinases, which are well known for their ability to recognize and phosphorylate agonist activated G protein-coupled receptors (GPCRs), resulting in their desensitization^5^. In particular, GRKs act as crucial negative regulators of variety of GPCRs, including adrenergic, muscarinic, dopamine, and chemokine receptors^5^. There are seven subtypes of GRKs (GRK1−7) that have been identified to date, which are subdivided into three classes based on the sequence homology: rhodopsin kinases subfamily (GRK1 and GRK7), the β-adrenergic receptor kinases subfamily (GRK2/GRK3), and the GRK4 subfamily (GRK4, GRK5, and GRK6)^5–7^. Expression of GRK1 and GRK7 is specific to the retina^8^ and expression of GRK4 is limited to the testis^9^. In contrast, GRK2, GRK3, GRK5, and GRK6 isoforms are widely distributed in multiple cell types^10^.

Several studies showed that the expression and activity of GRKs are impaired in many pathological conditions^11–16^, and even cancer. Specifically, recent studies have demonstrated that GRK5 is required for cancer cell cycle progression^17,18^. GRK5 also regulated p53 phosphorylation and degradation, leading to inhibition of apoptosis in reponse to DNA damage in cultured osteosarcoma cells and in mice^19^. Furthermore, inactivation of GRK5 reduced growth, invasion, and metastasis of human prostate cancer through directly phosphorylating cytoskeletal-membrane attachment protein moesin^20^. GRK5 was identified to phosphorylate nucleophosmin (NPM1), therefore regulating sensitivity to polo-like kinase inhibitor-induced apoptosis of breast cancer cells^21^, and its upregulation mediated by tazarotene-induced gene 1 (TIG1) significantly suppresses colon cancer growth^22^. However, it is still unclear whether GRK5 plays any role during NSCLC tumor progression.

In this study, we explored the putative functional roles of GRK5 in NSCLC, and found that GRK5 protein expression in NSCLC cancerous tissues was higher than that in non-cancerous normal tissues, and the GRK5 high expression NSCLC patients had significantly worse survival rate than low expression patients. In addition, we demonstrated that knockdown of GRK5 inhibited NSCLC cell proliferation, migration in vitro, and xenograft tumor formation in vivo by promoting cell cycle arrest at G2/M phase and inducing cellular apoptosis. These findings indicate a critical role of GRK5 in the cure and prognosis of NSCLC, which provide a possible new direction for NSCLC research in future.

## Results

### GRK5 expression in NSCLC

**Fig. 1:**
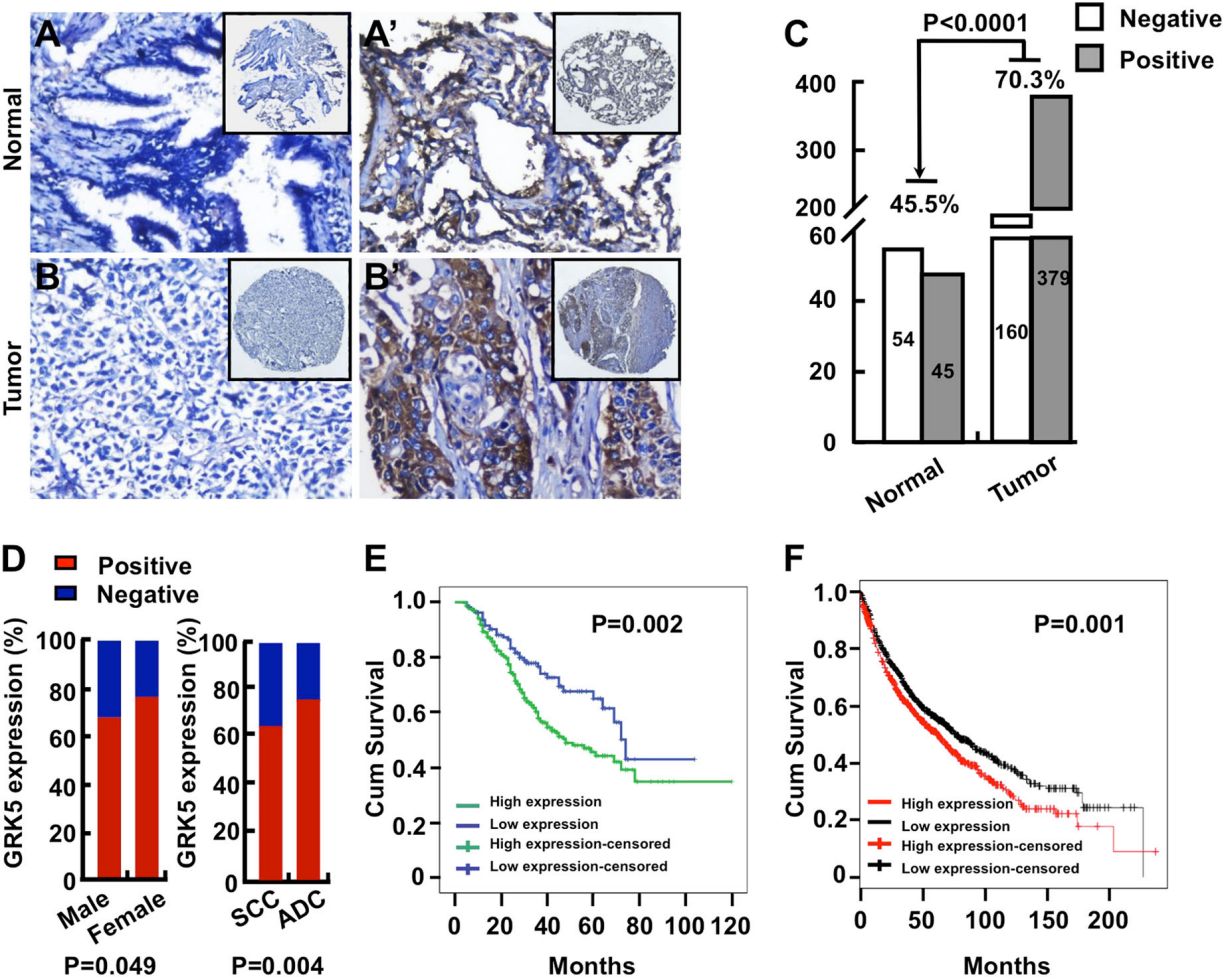
GRK5 is highly expressed in NSCLC cancerous tissues. **a**,** b′** NSCLC tissue microarrays (TMAs) contained sections of 539 NSCLC cancerous tissues and 99 normal non-cancerous lung tissues were stained by GRK5 antibodies. Representative images with differential magnification (**a** and **b**×200; **a′** and **b′**×200) of IHC staining were shown. **a**-**a′** Representative images of both negative and positive GRK5 expression in normal lung tissues were shown. **b**-**b′** Representative images of both negative and positive GRK5 expression in NSCLC tumor tissues were shown. **c** Quantification data for IHC staining result, the GRK5-positive staining percentages in both normal and NSCLC tissues were shown. *p* < 0.0001. Normal: normal lung tissues; Tumor: NSCLC cancerous tissues. **d** The percentage for GRK5-positive staining was higher in female and ADC NSCLC cancerous tissues, respectively. **e**, **f** Kaplan−Meier plotter was used to analyze the protein expression data from IHC (**e**) and transcriptional RNA data (**f**). GRK5 high expression in NSCLC had a significantly worse survival rate than low expression patients. Different color lines correlated with different GRK5 protein (**e**; *p* = 0.002) or RNA (**f**; *p* = 0.001) expression levels

Among all the GRKs, GRK5 is one of the most studied that has been indicated to be involved in many pathologic conditions^23^. GRK5 not only preferentially binds phospholipids and displays membrane localization, but also is able to localize into nucleus^24^. Until now, the effects of GRK5 on cancer progression are controversial depending on its subcellular localization and the cancer type^23^. In order to examine the functional roles of GRK5 in NSCLC, we firstly investigated the correlation between the expression of GRK5 protein and clinicopathological features of NSCLC, and applied immunohistochemistry (IHC) in the NSCLC tissue microarray (TMA) (Fig. 1a–c) that contained 539 NSCLC cancerous tissues (including 248 lung SCC and 291 ADC) and 99 normal or non-cancerous tissues (hereafter referred to as normal tissues). All specimens were validated by the pathologist and graded using the pathologic and clinic stage (Table 1). We found that the percentage of positive intensive GRK5 expression is significantly higher in NSCLC cancerous tissues (70.3%; 379/539) than that in normal lung tissues (45.5%; 45/99), also higher in ADC (75.3%; 219/291) and female (76.1%; 108/142) cancerous tissues than that in SCC (64.5%; 160/248) and male (68.3%; 271/397) cancerous tissues, respectively (Fig. 1d). However, we did not observe that GRK5 mis-expression correlated with age or TNM stages in NSCLC TMA (data not shown). We also analyzed the association of GRK5 protein expression with overall survival, and found that GRK5 high expression in NSCLC had a significantly worse overall survival rate than low expression patients (Fig. 1e). In addition, by applying Kaplan−Meier plotter (http://kmplot.com), which contains the Affymetrix gene expression data set for 1926 lung cancer patients, we also found that GRK5 high expression negatively correlates with worse overall survival rate (Fig. 1f)^25^.

**Table 1:** Clinicopathological characteristics of patients with non-small cell lung cancer (NSCLC) and non-cancerous lung tissues in the tissue arrays

Patients characteristics	No. of patients (%)
**NSCLC**	
*Age(years)*	
≤50	242 (44.9)
>50	297 (55.1)
*Gender*	
Male	397 (73.7)
Female	142 (26.3)
*Clinical stages*	
Stage I	125 (23.2)
Stage II	148 (27.5)
Stage III	239 (44.3)
Stage IV	27 (5.0)
*Lymph node status*	
N0	208 (38.6)
N1/N2/N3	331 (61.4)
*Histological type*	
SCC	248 (46.0)
ADC	291 (54.0)
*Differentiation*	
Well	34 (6.3)
Moderate	240 (44.5)
Poor	265 (49.2)
*Survival status*	
Alive	346 (64.2)
Death	193 (35.8)
**Non-cancerous lung tissues**	
*Age(years)*	
≤50	82 (78.8)
>50	22 (21.2)
*Gender*	
Male	60 (57.7)
Female	44 (42.3)

### GRK5 is highly expressed in NSCLC cancerous cell lines

**Fig. 2:**
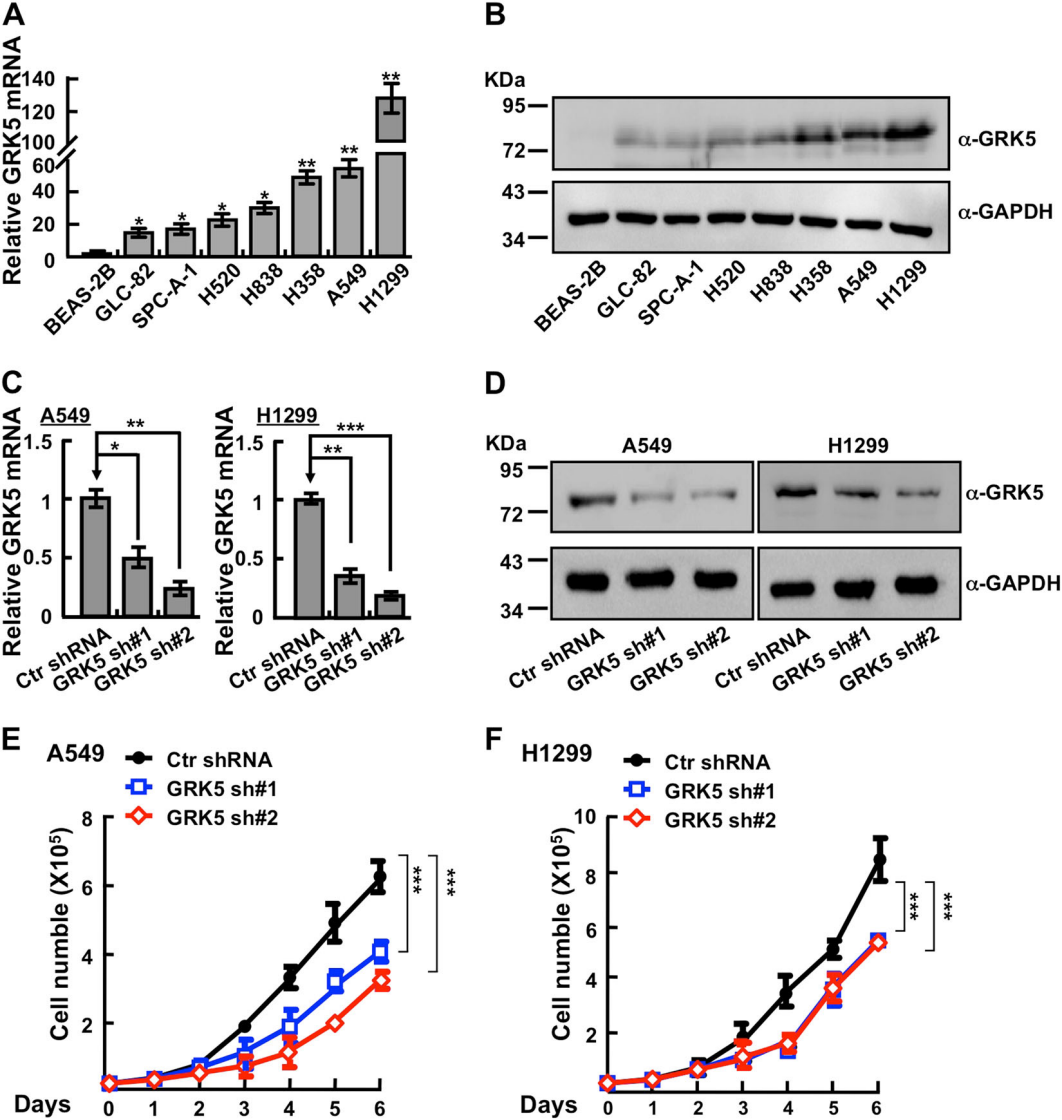
Depletion of GRK5 inhibits NSCLC cell proliferation. **a**, **b** Relative GRK5 mRNA (**a**) and protein (**b**) expressions in normal human bronchial epithelial cells (BEAS-2B) and NSCLC cell lines (GLC-82, SPC-A-1, H520, H838, H358, A549, and H1299). BEAS-2B was used as control. Indicated cell lysates were probed with GRK5 and GAPDH antibodies, respectively. Representative images were shown. **c**, **d** Individual GRK5 shRNA knockdown efficiency in A549 and H1299 was verified by real-time PCR (**c**) and western blot (**d**). Scramble shRNA was set as control. Ctr: scramble shRNA control; sh#1: shRNA#1; sh#2: shRNA#2. α: antibody. **e**, **f** GRK5 knockdown significantly inhibited A549 (**e**) and H1299 (**f**) cell proliferation. *p < 0.05, **p < 0.01, ***p < 0.001, *t*-test

To examine whether GRK5 is also highly expressed in NSCLC cancerous cells, we analyzed the relative mRNA and protein expressions of GRK5 in normal human bronchial epithelium cell line (BEAS-2B) and NSCLC cancerous cell lines (GLC-82, SPC-A-1, H520, H838, H358, A549, and H1299). Consistent with our findings in clinical cancerous tissues, we found that both GRK5 mRNA and protein expressions are unanimously upregulated in all NSCLC cancerous cell lines, with relative higher expression levels in A549 and H1299 cells (Fig. 2a, b). Owing to the higher expression of GRK5 in the above cancerous cell lines, we speculated that GRK5 might also be frequently mutated in human NSCLC cancers. Consistent with our hypothesis, we identified many GRK5 mutations in NSCLC by applying the cBioPortal web resource^26^ (Table 2), indicating that GRK5 plays an important role in lung homeostasis.

**Table 2:** GRK5 mutation information in human NSCLC cancers

Sample ID	Cancer study	AA change	Type
LUAD-CHTN-3090416	Lung adeno (Broad)	A292V	Missense
LUAD-YINHD	Lung adeno (Broad)	E254D	Missense
TCGA-05-4410-01	Lung adeno (TCGA pub)	C42*	Nonsense
TCGA-05-4417-01	Lung adeno (TCGA pub)	P314S	Missense
TCGA-38-4631-01	Lung adeno (TCGA pub)	R399L	Missense
TCGA-44-4112-01	Lung adeno (TCGA pub)	L77M	Missense
TCGA-50-5932-01	Lung adeno (TCGA pub)	V308I	Missense
TCGA-64-5778-01	Lung adeno (TCGA pub)	S590I	Missense
TCGA-05-4410-01	Lung adeno (TCGA)	C42*	Nonsense
TCGA-05-4417-01	Lung adeno (TCGA)	P314S	Missense
TCGA-38-4631-01	Lung adeno (TCGA)	R399L	Missense
TCGA-44-4112-01	Lung adeno (TCGA)	L77M	Missense
TCGA-50-5932-01	Lung adeno (TCGA)	V308I	Missense
TCGA-64-5778-01	Lung adeno (TCGA)	S590I	Missense
TCGA-18-3409-01	Lung squ (TCGA pub)	S114S	splice_region
TCGA-66-2763-01	Lung squ (TCGA pub)	K522E	Missense
TCGA-18-3406-01	Lung squ (TCGA pub)	G198V	Missense
TCGA-37-3789-01	Lung squ (TCGA pub)	G459W	Missense
TCGA-66-2763-01	Lung squ (TCGA)	K522E	Missense
TCGA-18-3409-01	Lung squ (TCGA)	S114=	splice_region
LUAD-YINHD-Tumor	NSCLC (TCGA 2016)	E254D	Missense
TCGA-05-4410-01	NSCLC (TCGA 2016)	C42*	Nonsense
TCGA-05-4417-01	NSCLC (TCGA 2016)	P314S	Missense
TCGA-38-4631-01	NSCLC (TCGA 2016)	R399L	Missense
TCGA-44-4112-01	NSCLC (TCGA 2016)	L77M	Missense
TCGA-50-5932-01	NSCLC (TCGA 2016)	V308I	Missense
TCGA-64-5778-01	NSCLC (TCGA 2016)	S590I	Missense
TCGA-18-3409-01	NSCLC (TCGA 2016)	S114S	splice_region
TCGA-66-2763-01	NSCLC (TCGA 2016)	K522E	Missense
LUAD-RT-S01301-Tumor	NSCLC (TCGA 2016)	G195W	Missense
TCGA-21-1079-01	NSCLC (TCGA 2016)	V116I	Missense
TCGA-21-1083-01	NSCLC (TCGA 2016)	G269C	Missense
TCGA-33-AASL-01	NSCLC (TCGA 2016)	E156K	Missense
TCGA-44-2656-01	NSCLC (TCGA 2016)	T485S	Missense
TCGA-44-2668-01	NSCLC (TCGA 2016)	K475*	Nonsense
TCGA-58-A46K-01	NSCLC (TCGA 2016)	R216L	Missense
TCGA-77-8145-01	NSCLC (TCGA 2016)	K210I	Missense
TCGA-77-A5GF-01	NSCLC (TCGA 2016)	G488V	Missense
TCGA-85-7696-01	NSCLC (TCGA 2016)	R23L	Missense
TCGA-85-A512-01	NSCLC (TCGA 2016)	K564N	Missense
TCGA-99-8025-01	NSCLC (TCGA 2016)	M515I	Missense

### Depletion of GRK5 inhibits NSCLC cell proliferation and xenograft tumor formation

**Fig. 3:**
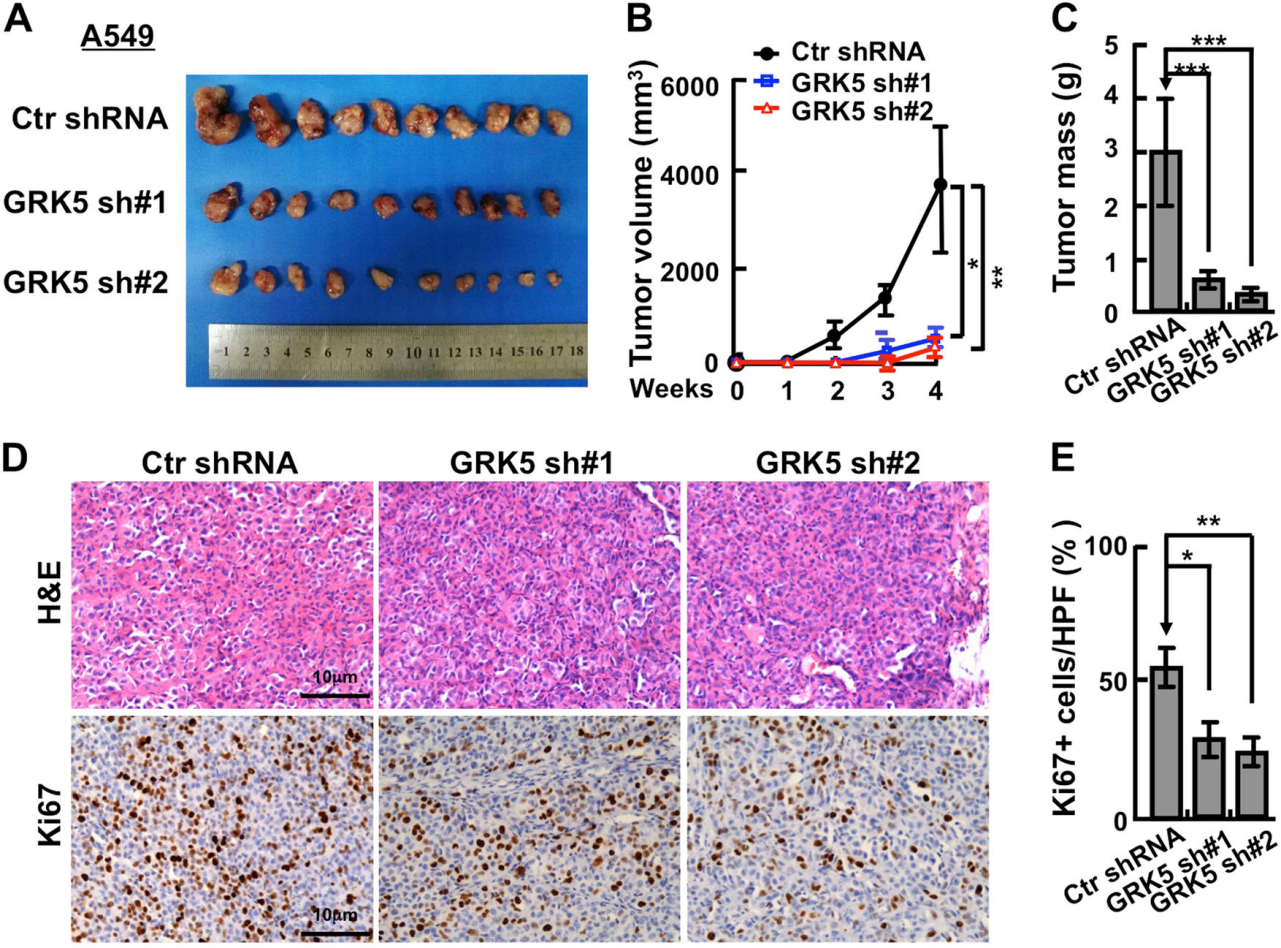
Knockdown of GRK5 inhibits xenograft tumor formation in vivo. **a** Representative xenograft tumors for indicated cells were shown. **b** GRK5 knockdown significantly reduced xenograft tumor growth in male nude mice by tumor volume examination. **c** Depletion of GRK5 significantly suppressed xenograft tumor weights. **d** Representative images of H&E and Ki67 IHC for xenograft tumor sections. Scale bar: 10 μm. **e** Quantification data for (**d**). HPF high power field. *p < 0.05, **p < 0.01, ***p < 0.001, *t*-test

We then picked NSCLC cancerous cell lines A549 and H1299 to further validate the functional roles of GRK5. We inhibited GRK5 mRNA expression with two independent lenti-viral shRNAs (Materials and Methods, Supplementary Table 1), and verified the inhibition efficiency by comparing both relative mRNA and protein expression levels in GRK5-shRNA-targeted cell lines with that in scramble shRNA control cells (Fig. 2c–d). As expected, stable knockdown of GRK5 inhibited A549 and H1299 cell proliferation in vitro (Fig. 2e, f). To further verify whether GRK5 is important for NSCLC progression in vivo, we performed a tumorigenesis assay in nude mice. Fifteen male nude mice aged 5 weeks were randomly divided into three groups, and then injected with A549 cells stably expressing scramble control shRNA or two GRK5-targeting shRNAs, respectively (5×10^6^ cells/point subcutaneously). Mice were monitored each day and tumor growth was measured every 3 days. As expected, the A549 cell proliferation and tumor formation in vivo was dramatically retarded compared with that of scramble shRNA-transformed cells, and both tumor weights and volumes in the A549 GRK5 knockdown cells were less than those of A549-scramble shRNA cells (Fig. 3a–c). In addition, the Ki67 IHC staining in the xenograft tumor tissues showed that the GRK5 knockdown indeed inhibited cancerous cell proliferation in vivo (Fig. 3d, e).

### GRK5 regulates NSCLC cell migration, cell cycle, and apoptotic signaling pathway

**Fig. 4:**
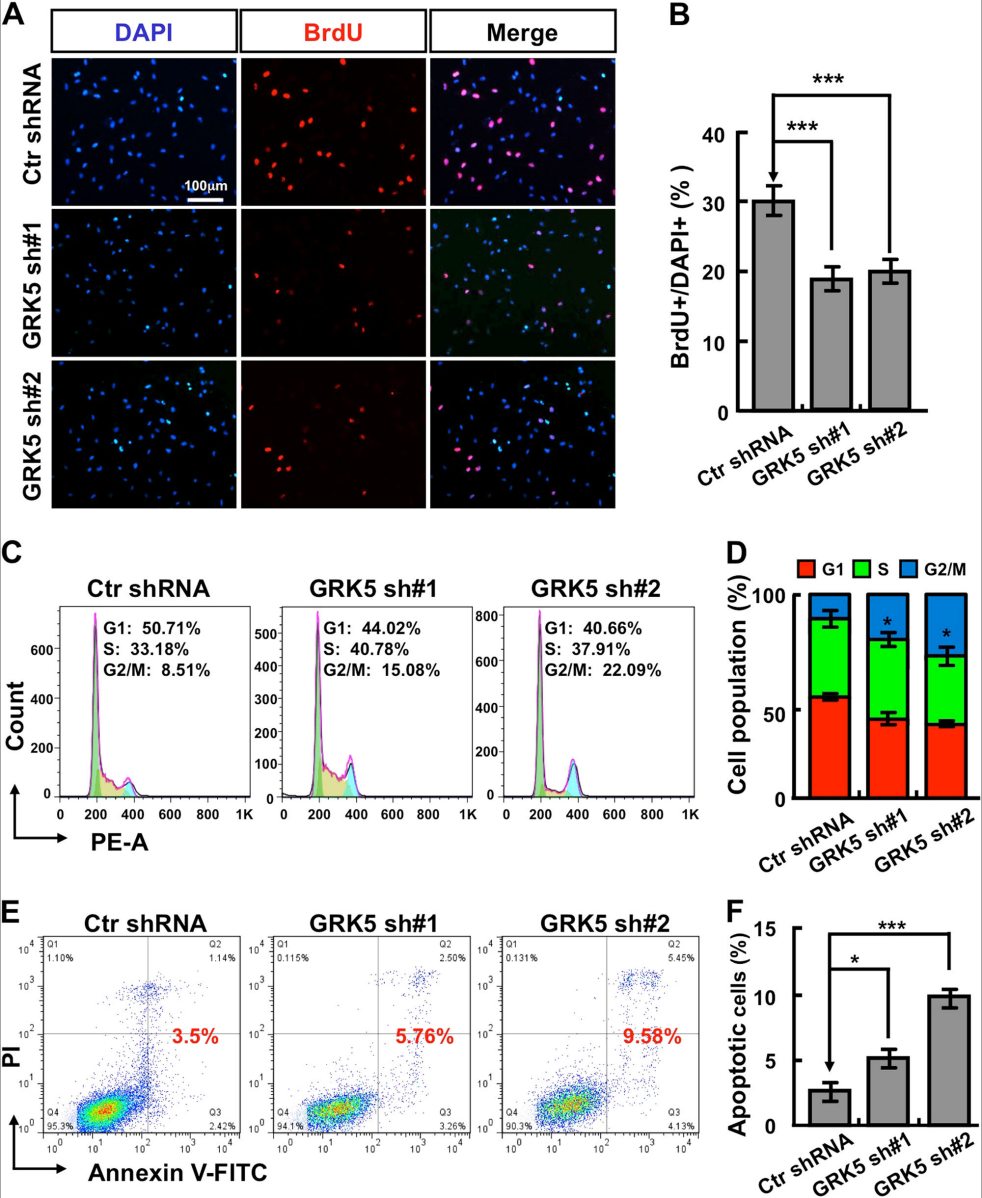
GRK5 inhibition promotes A549 cell cycle arrest and cellular apoptosis. **a** GRK5 knockdown inhibited DNA synthesis in A549 cells by BrdU incorporation assay. Scale bar: 100 μm. **b** Quantification data for (**a**). **c** Depletion of GRK5 increased G2/M cell population by FACS analysis. Indicated cells were stained with PI for analyzing cell cycle distribution. **d** Quantification data for (**c**). **e** Indicated cells were collected for Annexin V staining and flow cytometry assay. **f** Quantification data for (**e**). *p < 0.05, **p < 0.01, ***p < 0.001, *t*-test

**Fig. 5:**
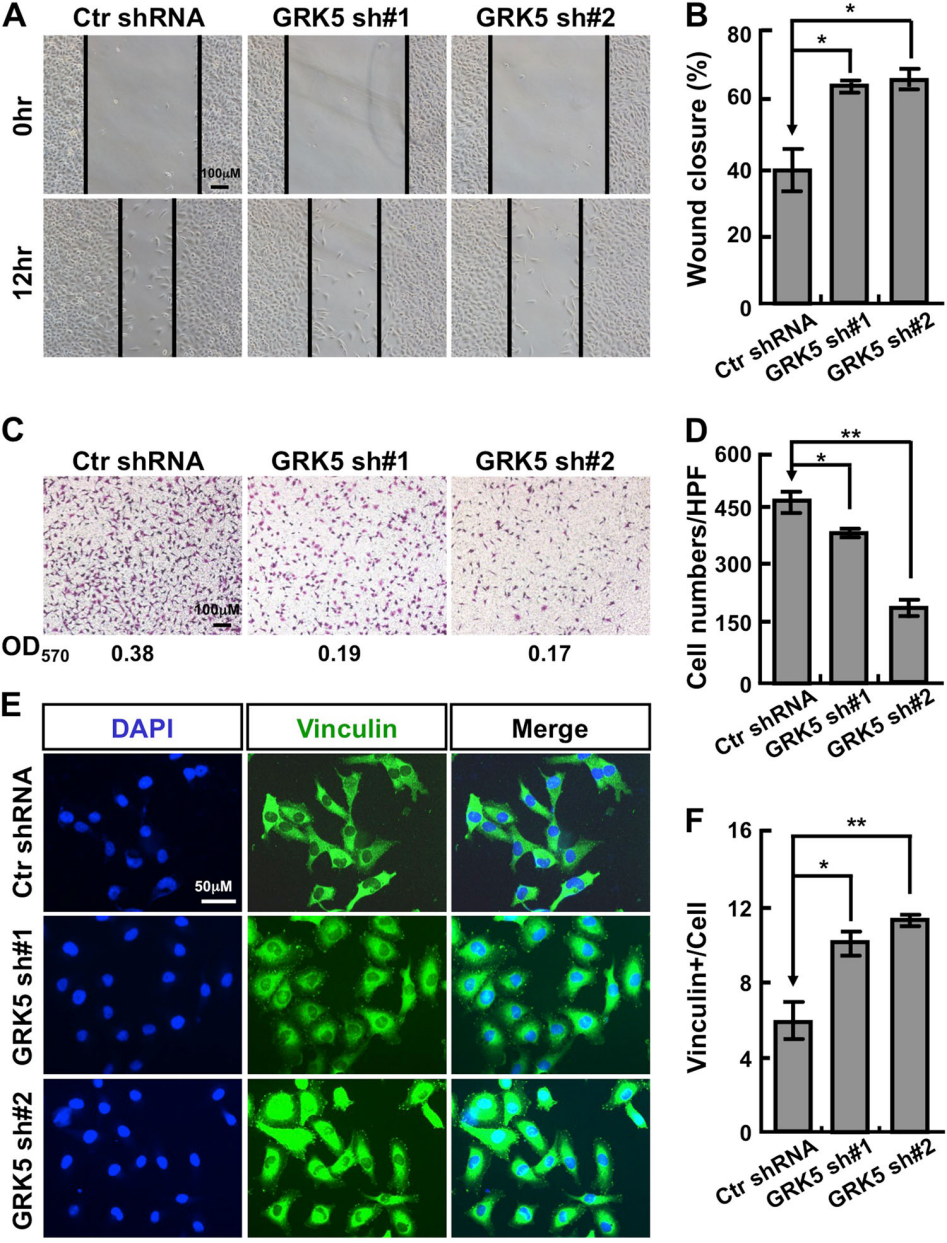
GRK5 knockdown inhibits A549 cell migration. **a** Wound-healing assay showed that knockdown of GRK5 inhibited the migration of A549 cells. Scale bar: 100 μm. **b** Relative width change (wound closure) comparing the 12 h width with 0 h starting width (%), quantification data for (**a**). **c** Knockdown of GRK5 decreased the migration ability of A549 by transwell assay. The OD_570_ number was indicated below for indicated images. Scale bar: 100 μm. **d** Quantification data for (**c**). **e** GRK5 depletion dramatically promoted vinculin staining positive numbers. Scale bar: 50 μm. **f** Quantification data for (**c**). *p < 0.05, **p < 0.01, ***p < 0.001, *t*-test

Since knockdown of GRK5 inhibited A549 and H1299 cell proliferation (Fig. 2e, f), we hypothesized that GRK5 might be important for NSCLC cell cycle distribution as well. Indeed, we observed that GRK5 knockdown significantly decreased the ratio of BrdU-positive cells in A549 and H1299 (Fig. 4a, b and Fig. S1A−B). We then characterized the cell cycle transition by flow cytometry analysis, and found that GRK5 knockdown increased the ratio of G2/M cell population from 8.51% in control cells to 15.08% or 22.09%, respectively in independent GRK5-targeting shRNA knockdown A549 cells (Fig. 4c, d and Fig. S1C−D). To figure out whether the G2/M cell cycle arrest leads to cellular apoptosis by depleting GRK5, cells were collected for annexin V staining and flow cytometry assay. Consistent with the above findings, the percentages of apoptotic cells in GRK5 knockdown cells were significantly higher than that in scramble shRNA control cells (Fig. 4e, f and Fig. S1E−F). These data indicated that GRK5 is important for both NSCLC cell cycle transition and apoptotic signaling. Furthermore, we decided to test whether GRK5 regulates NSCLC cell migration, and performed both wound healing and transwell assays. As the data show, GRK5 depletion in both A549 and H1299 dramatically decreased cell migration (Fig. 5a–d and Fig. S2A−D). Consistent with the migration result and former findings^20^, staining with antibody against vinculin, one of the commonly used markers for focal adhesion, showed that cells markedly increased their focal adhesions along the cell membrane after GRK5 knockdown in both A549 and H1299 cells (Fig. 5e, f and Fig. S2E-F).

In summary, our data indicated that GRK5 might play as an oncogenic factor in NSCLC, which could be used as a novel candidate biomarker. However, further studies should be carried out using GRK5 alone or in combination with other NSCLC specific biomarkers to develop novel NSCLC therapeutic strategies.

## Discussion

It has been demonstrated that GRK5 plays pivotal roles in differential pathologic conditions. GRK5 attenuates atherosclerosis through desensitizing tyrosine kinase receptors, such as CSF-1R and PDGFR^16^; GRK5 is upregulated in mycocardium leading to reduced cardiac β-AR responsiveness in failing human heart^27^; overexpression of GRK5 enhances the internalization of glucagon receptor mediating hyperglycaemic effects of glucagon in diabetes^28^; GRK5 has also been indicated to be involved in Parkinson disease^29^. GRK5 shuttles between the cytosol and the nucleus to regulate intracellular signaling depending on cell type, stimuli, and kinase subcellular localization; the activities of HDAC and NFκB are also regulated by GRK5^30,31^. GRK5 maps on chromosome 10 at q24 region, chromosome translocation of which has been frequently observed in thyroid or glia tumors^32^, suggesting that alteration of GRK5 could be involved in certain cancers. Several studies indicated that GRK5 functions as negative regulator in some types of cancers. For example, it has been demonstrated that GRK5 low expression is associated with TSH receptor-stimulated high level of cAMP in several human thyroid cancer types (DTC)^33^; TIG-1 inhibits colon cancer cell HCT116 proliferation by inducing GRK5 expression^34^; co-expression of GRK5 inhibits Kaposi’s sarcoma cell proliferation induced by constitutive activation of human herpesvirus -8 (KSHV) G protein-coupled receptor^35^. Besides the inhibitory effect of GRK5 on tumor growth, GRK5 is also able to promote cancer cell proliferation. GRK5 phosphorylates p53 promoting its degradation, which inhibits p53-mediated cellular apoptosis in osteosarcoma^19^; GRK5 colocalizes with and phosphorylates moesin to promote actin remodeling, invasion, and metastasis of PC3 cell^20^.

In this study, for the first time, we characterize the functional roles of GRK5 in NSCLC. We found that GRK5 is highly expressed in NSCLC cancerous tissues compared with normal non-cancerous tissues, whose high expression in NSCLC patients correlates with worse prognosis and survival rate than low expression patients, and is frequently mutated in NSCLC cancer tissues. We then examined the protein and mRNA expression of GRK5 in NSCLC cancerous cell lines comparing with normal human bronchial epithelium cells BEAS-2B, and found that GRK5 is unanimously upregulated in cancerous cells. Depletion of GRK5 inhibits A549 and H1299 cell proliferation, migration in vitro, and xenograft tumor formation in vivo. Furthermore, we provided evidence showing that GRK5 knockdown promotes cell cycle arrest at G2/M phase and cellular apoptosis. The above data indicate that GRK5 functions as an oncogenic factor in NSCLC, although the mechanism by which GRK5 regulates NSCLC cell proliferation, migration, cell cycle, and xenograft tumor formation is not clear yet. Consistent with the phenotype observed in PC3 for GRK5^20^, the vinculin staining positive numbers are dramatically increased in GRK5-depleted NSCLC cells, which partially explains the reduced cell migration ability. Since GRK5 also regulates HDAC and NFκB, it will be important to further validate the possible phenomenon in NSCLC cells to explore the underlying mechanisms.

Based on the above multiple roles of GRK5 in many pathological conditions, it has become one of the promising therapeutic GRKs target to treat human diseases in future. However, to date, no effective and specific inhibitor against GRK5 has been designed and synthesized for antagonizing its oncogenic effect. On the other hand, given the anti-tumor effect of GRK5, exploring the putative compound or other molecular events that induce the upregulation of GRK5 could be more effective.

In summary, GRK5 is involved in the development and progression of several human diseases including cancer, although its role in tumorigenesis is still complex and ambiguous based on its opposite effects, cancer types, kinase activity, and protein subcellular localization. At this time, there is a lack of gene expression data derived from NSCLC patients that supports its high level expression is important for NSCLC progression; further studies will be needed to better define the functional mechanisms.

## Materials and methods

### Tissue microarrays and clinical data

In this study, NSCLC patients were subjected to surgical treatment at the Department of Thoracic Surgery at the Second Xiangya Hospital of Central South University (Changsha, China) from 2003 to 2013. All tumor samples and non-cancerous lung tissues were obtained from the Department of Pathology, the Second Xiangya Hospital of Central South University. These patients had been subjected to routine staging and definitive surgical resection of the lung and systematic mediastinal lymph node dissection. All patients had a confirmed histological diagnosis of NSCLC according to WHO histological classification of lung cancer. The staging classification of the current analysis was carried out based on the criteria of the 7th edition of the AJCC/UICC TNM staging system of lung cancer (2009). No patients had been previously treated with chemotherapy and radiotherapy at the time of original operation. Complete clinical record and follow-up data were available for all patients. Overall survival time was calculated from the data of diagnosis to the date of death or the data last known alive. Immunohistochemical staining of TMA sections were scored independently by SZ and SF blinded to the clinicopathological data, at ×200 magnification light microscopy. The evaluation was based on the staining intensity and extent of staining. Staining intensity for GRK5 was scored as 0 (negative), 1 (weak), 2 (moderate), and 3 (strong). Staining extent was scored as 0 (<10%), 1 (1–25%), 2 (26–50%), 3 (>50%), depending on the percentage of positive-stained cells. The sum of the staining intensity and the staining extent scores ranged from 0 to 6. An optimal cut-off point was identified as follows: a staining index score of 0–2 was used to define tumors with negative expression and 3–6 indicated positive expression of GRK5 protein. Agreement between the two evaluators was 95%, and all scoring discrepancies were resolved through discussion between the two evaluators. Kaplan−Meier analysis was performed for overall survival curves and statistical significance was assessed using the log-rank test. Cox proportional hazard regression model was used to estimate the independent prognostic factor GRK5 protein. Two-sided statistical analysis was used and the data were considered to be statistically significant when *p* < 0.05.

### Constructs and cell culture

Independent shRNAs targeting to GRK5 were constructed using pLKO.1 vector; constructs were sequence verified. The detailed oligo information and the primers sequences are indicated in Supplementary Table 1. Lenti-viruses were generated according to the manufacturer’s protocol; cells were infected by viruses twice for 48 and 72 h with viral supernatants containing 4 μg/ml polybrene. HEK-293T cells were obtained from ATCC and cultured following standard protocol. BEAS-2B cell line was a gift from Dr. Hong-Bin Ji at SIBS, CAS, China. GLC-82, SPC-A-1, H520, H838, H358, and A549 cells were all cultured in RPMI-1640 medium (Hyclone) supplemented with 10% fetal bovine serum (FBS) and 1% penicillin/streptomycin, and H1299 cells were cultivated in DMEM (Hyclone). All cells were cultured at 37 °C in a 5% CO_2_ humidified environment.

### Western blotting

Indicated cells were lysed with RIPA buffer, supernatants were boiled at 100 °C for 5 min, samples were separated by electrophoresis and transferred to polyvinylidene fluoride membrane (Millipore). Primary antibodies used in this study are: GRK5 (Santa Cruz Biotechnology, Cat# sc-565, dilution 1:1000) and GAPDH (Proteintech Group, Cat# 60004-1-Ig, 1:5000). The secondary antibodies conjugated with horseradish peroxidase goat anti-mouse IgG (Proteintech Group, Cat# SA00001-1, dilution 1:3000) or horseradish peroxidase-cojugated goat anti-rabbit IgG (Jackson ImmunoResearch, Cat# 111-035-144, dilution 1:2000), followed by analyzing with the MiniChemi imaging systems (Beijing, China).

### Cell proliferation, migration, BrdU incorporation, and FACS assays

Indicated 20,000 cells/well were plated into 12-well plates. Cells per well in triplicate for each day were then trypsinized and determined using automatic cell analyzer countstar (Shanghai Ruiyu Biotech Co., China, IC 1000). The migration ability of indicated cells was evaluated by wound healing and transwell assays. For wound healing assay, cells were plated in six-well plates at 1×10^6^ cells/well in 2 ml of culture medium. Twenty-four hours later, a wound was scratched in the adherent cell monolayers with an Eppendorf tip. Wounds were imaged at 5–10 positions along each well. For transwell assay, indicated cells in serum-free medium were plated on uncoated insets and incubated using 24-well chemotaxis chambers (Corning cell culture inserts, 8 μm pore size). Fetal bovine serum (10%) was added to the bottom wells of the chambers as chemo-attractant. Cells were allowed to migrate through the membrane for indicated time. The non-migrating cells were removed and migrating cells to the lower face were stained with cresyl violet (Sigma). Stained cells in the entire fields were counted under an inverted microscope. Cells were grown in serum containing media for 24 h. Twenty minutes prior to fixation, cells were treated with 10 µm BrdU (Abcam, Cat# ab142567, dilution 1:100), then fixed with 4% PFA for 20 min. Cells were stained with BrdU (Cell Signalling Technology, Cat# 5292 s, dilution 1:1400) primary antibody followed by secondary antibody (Abclonal, Cat# 61303, dilution 1:500). Cell nuclei were stained with DAPI. Five randomly selected fields/sample were imaged on a Nikon Ti fluorescence microscope and quantified. For cell cycle assays, fixed cells were washed by cold phosphate-buffered saline, stained by PI for 30 min at 37 °C and then analyzed by flow cytometer. For apoptosis assays, cell suspensions in binding buffer were stained for Annexin V (BD Pharmingen) and PI, respectively. At least 10,000 events were recorded for each replicate and analyzed using FlowJo software.

### Xenograft tumor formation assay

A total of 15 male nude mice at 4–5 weeks of age were purchased from Shanghai SLAC Laboratory Animal Co., Ltd (Shanghai, China) and randomly divided into indicated groups, and injected subcutaneously with indicated cell lines; each experiment was repeated at least three times. One week post-injection, tumor sizes in all groups were measured with Vernier calipers (Suzhou, China) every 3 days for 4 weeks. All mice were killed at the end of the experiment and tumors were harvested and weighed. Tumor volumes were calculated by the formula length × (width)^2^/2. A portion of each tumor tissue was fixed in 4% paroformaldehyde and embedded in paraffin for IHC analysis.

### Immunohistochemical

Formalin-fixed and paraffin-embedded tissue specimens were sliced into 4 µm sections and then deparaffinized in xylene, rehydrated through graded ethanol solutions and immersed in a 3% hydrogen peroxide solution in methanol for 20 min to inhibit endogenous peroxidase activity. After washing with Tris buffer saline (TBS), antigen retrieval was performed by placing the slides at 95 °C for 20 min in a microwave oven and allowed to cool for 1 h at room temperature. The slides were again washed three times with TBS, and nonspecific binding was blocked by pre-incubation with 10% goat serum for 20 min at room temperature. Slide was then incubated for 1 h at room temparature with primary anti-ki67 (Fuzhou Maixin Biotech, Cat# RMA-0542) or GRK5 antibody (Santa Cruz Biotechnology, Cat# sc-565, dilution 1:200). After washing the slides three times with TBS, sections were subsequently treated with HRP-labeled second antibody (DAKO, Danmark) for 40 min. Diaminobenzidine was used as a chromogen followed by hematoxylin counterstaining. The slides were then dehydrated, cleared with xylene, and mounted.

### Quantitative real-time PCR

Total RNA was extracted from indicated cell lines using RNAiso Plus (Takara, Beijing, China, Cat# 108-95-2) and reverse transcribed using a PrimeScript RT reagent Kit (Takara Bio, Beijing, China, Cat# RR047A). One microgram cDNA was subjected to qRT-PCR using FastStart Universal SYBR Green Master Mix (Roche, Cat# 04194194001) to detect expression levels. Reactions were performed in triplicate using Applied Biosystems 7500 machine. GAPDH was used to normalize expression level.

### Immunofluorescence staining

Cells were fixed with 4% paraformaldehyde for 20 min and permeabilized with PBS plus 0.1% Triton X-100, and blocked by PBS with 10% normal goat serum. Cells were then incubated with the vinculin (Sigma-Aldrich, Cat# V9131, 1:400) primary antibody at 4 °C overnight, and probed with fluorescence-conjugated secondary antibodies (Abclonal, Cat# AS001, 1:500) at room temperature for 2 h. Nuclei were stained by DAPI and the images were analyzed.

### Statistical analyses

Data are represented as mean ± SEM, and error bars also indicate SEM. *p* values were calculated by either unpaired or paired two-tailed Student’s *t* test, *p < 0.05, **p < 0.01, and ***p < 0.001. All analyses were performed using GraphPad Prism software (GraphPad Software, Inc.). All the experiments were performed at least three independent times.

## Electronic supplementary material

Supplementary Table 1

Supplementary Figure S1

Supplementary Figure S2

Supplementary Figure legend
