# Supplementary material for: GRK5 functions as an oncogenic factor in non-small-cell lung cancer
Source: Cell Death Dis. 2018 Feb 20;9(3):295. doi: 10.1038/s41419-018-0299-1 (PMC5833409; doi:10.1038/s41419-018-0299-1)
Supplement: Supplementary file 1 — Supplementary Table 1 [file 41419_2018_299_MOESM1_ESM.docx]

**Supplementary Table 1. The sequences of primers used in this study.**

| Name | Sequences (5’-3’) |
| --- | --- |
| **Oligos for shRNAs** |  |
| Scramble shRNA_Forward Oligo | CCGGGCACTACCAGAGCTAACTCAGCTCGAG  CTGAGTTAGCTCTGGTAGTGCTTTTTG |
| Scramble shRNA_Forward Oligo | AATTCAAAAACACTACCAGAGCTAACTCAG  CTCGAGCTGAGTTAGCTCTGGTAGTGC |
| GRK5 shRNA#1_Forward Oligo | CCGGGCACAGTCTGTCCACGAGTACCTCGAG  GTACTCGTGGACAGACTGTGCTTTTTG |
| GRK5 shRNA#1_Reverse Oligo | AATTCAAAAAGCACAGTCTGTCCACGAGTAC  CTCGAGGTACTCGTGGACAGACTGTGC |
| GRK5 shRNA#2_Forward Oligo | CCGGCCGCCAGATCTGAACAGAAACCTCGAG  GTTTCTGTTCAGATCTGGCGGTTTTTG |
| GRK5 shRNA#2_Reverse Oligo | AATTCAAAAACCGCCAGATCTGAACAGAAAC  CTCGAGGTTTCTGTTCAGATCTGGCGG |
| **Primers for qPCR** |  |
| GRK5_Forward primer | CCTCCGAAGGACCATAGACA |
| GRK5_Reverse primer | GACTGGGGACTTTGGAGTGA |
| GAPDH_Forward primer | ACCACAGTCCATGCCATCAC |
| GAPDH_Reverse primer | TCCACCACCCCTGTTGCTGTA |
