# Supplementary figures and images for: GRK5 functions as an oncogenic factor in non-small-cell lung cancer
Source: Cell Death Dis. 2018 Feb 20;9(3):295. doi: 10.1038/s41419-018-0299-1 (PMC5833409; doi:10.1038/s41419-018-0299-1)

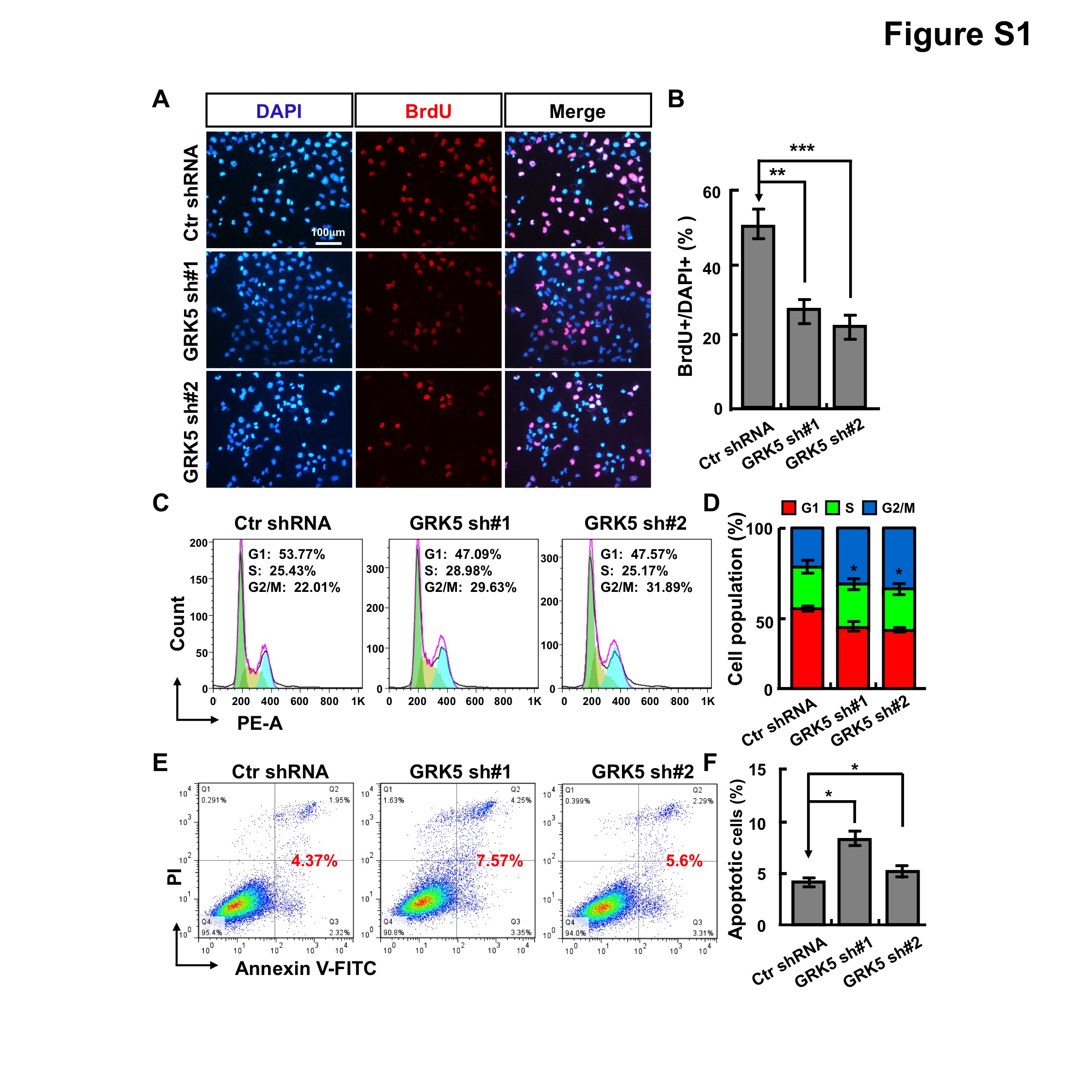

Supplement: Supplementary file 2 — Supplementary Figure S1 [file 41419_2018_299_MOESM2_ESM.jpg]

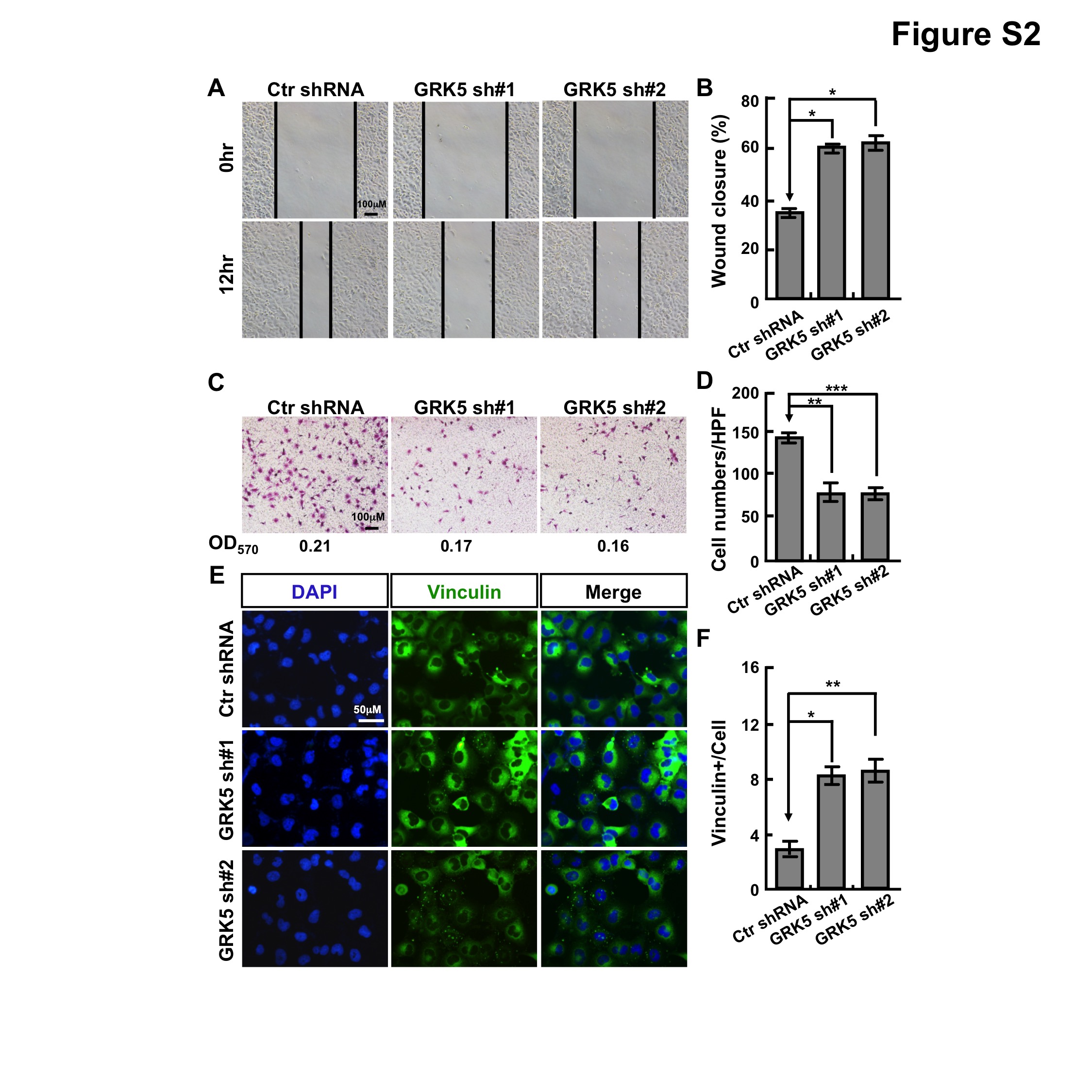

Supplement: Supplementary file 3 — Supplementary Figure S2 [file 41419_2018_299_MOESM3_ESM.jpg]
