# Supplementary material for: GRK5 functions as an oncogenic factor in non-small-cell lung cancer
Source: Cell Death Dis. 2018 Feb 20;9(3):295. doi: 10.1038/s41419-018-0299-1 (PMC5833409; doi:10.1038/s41419-018-0299-1)
Supplement: Supplementary file 4 — Supplementary Figure legend [file 41419_2018_299_MOESM4_ESM.doc]

**Supplementary figure legend**

**Figure S1: GRK5 inhibition promotes H1299 cell cycle arrest and cellular apoptosis. (A)** GRK5 knockdown inhibited DNA synthesis in H1299 cells by BrdU incorporation assay. Scale bar: 100μm. **(B)** Quantification data for (A). **(C)** Depletion of GRK5 increased G2/M cell population by FACS analysis. Indicated cells were stained with PI for analyzing cell cycle distribution. **(D)** Quantification data for (C). **(E)** Indicated cells were collected for Annexin V staining and flowcytometry assay. **(F)** Quantification data for (E). *p<0.05, **p<0.01, ***p<0.001, *t*-test.

**Figure S2: GRK5 knockdown inhibits H1299 cell migration.** **(A)** Wound-healing assay showed that knockdown of GRK5 inhibited the migration of H1299 cells. Scale bar: 100μm. **(B)** Relative width change (wound closure) comparing the 12hr width with 0hr starting width (%), quantification data for (A). **(C)** Knockdown of GRK5 decreased the migration ability of H1299 by transwell assay. The OD_570_ number was indicated below for indicated images. Scale bar: 100μm. **(D)** Quantification data for (C). **(E)** GRK5 depletion dramatically promoted vinculin staining positive numbers. Scale bar: 50μm. **(F)** Quantification data for (C). *p<0.05, **p<0.01, ***p<0.001, *t*-test.
